# Long-term trends in grain yield and main nutrient contents of three staple cereal crops in China (1980–2024)

**DOI:** 10.3389/fnut.2026.1936187

**Published:** 2026-09-07

**Authors:** Jinrui Bai, Minghui Hou, Guang Liang, Shui Yu, Baobao Shi, Jiazhang Huang, Haiquan Xu

**Affiliations:** Institute of Food and Nutrition Development, Ministry of Agriculture and Rural Affairs, Beijing, China

**Keywords:** grain yield, maize, nutrient contents, rice, wheat

## Abstract

**Background:**

Rice, wheat, and maize are China’s three main staple crops and key sources of dietary energy and essential nutrients. Decades of cultivar improvement and changes in field management have been accompanied by substantial increases in grain yield, while the nutrient composition of these crops may also have changed over. However, the long-term trends of these alterations are poorly understood. Characterizing these trends is crucial for safeguarding national food security and ensuring an adequate dietary nutrient supply for the Chinese population.

**Objective:**

To systematically characterize long-term trends in the grain yield and major nutrient contents of rice, wheat, and maize in China between 1980 and 2024. Methods: Data on rice, wheat, and maize from 1980 to 2024 were collected from publicly available food composition databases and published literature databases. Linear regression models were fitted for each crop and nutrient variable to characterize temporal trends in grain yield and nutrient concentrations.

**Results:**

From 1980 to 2024, grain yield overall increased in all three crops. Grain protein concentration declined significantly in rice, wheat, and maize. Fat content decreased significantly in wheat and maize, whereas no clear trend was detected for rice. Carbohydrate content decreased significantly in wheat and increased in maize; no clear trend was detected in rice. Dietary fiber content increased significantly in rice and wheat; no clear trend was detected in maize. Furthermore, grain yield was negatively associated with protein concentration across all three crops, and with fat concentration in wheat and maize, whereas yield was positively associated with dietary fiber concentration in rice and wheat.

**Conclusion:**

Grain yields of rice, wheat, and maize substantially increased in China from 1980 to 2024; however, long-term trends in nutrient composition differed among the three crops. These findings highlight the need to consider nutritional quality alongside yield in crop improvement and food security strategies, and provide important empirical evidence to inform food security policy and crop breeding programs.

**Systematic review registration:**

https://doi.org/10.17605/OSF.IO/G3CFH, Registration identifier: 10.17605/OSF.IO/G3CFH.

## 1 Introduction

China has traditionally consumed a cereal-based diet, and consequently, rice, wheat, and maize are the three main staple crops of the country. They form the foundation of China’s national food security and substantially contribute to dietary nutrient intake in the country. These cereals provide a substantial proportion of the Chinese population’s dietary energy and are crucial sources of protein, dietary fiber, vitamins, and minerals (1–4). Data from the 2015–2017 Chinese Nutrition and Health Survey revealed that the average cereal intake in the country was 305.8 g per standard person per day and that cereals account for approximately 51.5% of total energy intake and 46.9% of total protein intake. Since the 1980s, China’s agricultural production systems have undergone substantial changes in terms of their breeding priorities and field management practices (including fertilizer application, tillage, and crop rotation) (5, 6). These changes may have influenced the genetic characteristics of crops and the conditions under which they are grown, thereby affecting their nutrient compositions. Given that staple crops account for a substantial proportion of the Chinese diet, such compositional changes could affect dietary nutrient intake in the region. Hence, systematically characterizing these temporal trends is crucial for understanding their potential implications for food and nutrition security.

Research on China’s staple crops has generally focused on differences in quality traits across varieties, production regions, or growing conditions (7), with most studies emphasizing processing, appearance, eating quality, and cooking quality than long-term changes in nutritional composition. For example, Liu et al. reported that the head rice yield increased but the brown rice yield, grain length, and amylose content decreased among early-season rice cultivars released in China between 1987 and 2024 (8). Feng et al. compared 635 rice varieties released in China between 2000 and 2014 and discovered that amylose content decreased and gel consistency increased in indica hybrid rice (9); however, they did not examine long-term changes in other nutrient contents. In addition, several studies outside China have assessed historical changes in major components of cereal grains, including protein, fat, starch, and dietary fiber. Da Silva Volpato et al. analyzed maize hybrids in the United States from 1936 to 2018 and reported a decline in grain protein, fat, and dietary fiber content, along with an increase in starch content (10). Moreover, Ben Mariem et al. analyzed changes in wheat samples collected between 1850 and 2016 from 17 countries and reported an increase in carbohydrate content, together with a decrease in protein and mineral contents (11). El Hassouni et al. evaluated 282 European wheat cultivars registered during 1961–2020 and reported significant improvements in grain yield and resistance to disease and lodging, together with a slight decrease in grain mineral content (12).

Collectively, these studies demonstrate that cereal grain composition can change over time and that such changes may differ among crops and nutritional components. However, previous studies have generally focused on individual crops, specific nutrients. A comprehensive assessment of long-term changes in the major nutritional components of China’s three staple crops—rice, wheat, and maize—including protein, fat, carbohydrate, dietary fiber, and moisture contents—has not yet been conducted. Consequently, the direction, magnitude, and crop-specific patterns of nutritional changes in China’s major cereal crops remain unclear.

This study assessed temporal trends in the grain yield and nutrient composition of rice, wheat, and maize in China between 1980 and 2024. By integrating data from published studies, this analysis provides new insights into temporal trend in the nutritional composition of China’s major staple crops and improves understanding of how grain nutrient compositions have changed over recent decades. These findings provide new insights into the evolution of China’s cereal grain composition over recent decades and offer a basis for understanding potential implications for food and nutrition security.

## 2 Materials and methods

### 2.1 Data sources

This study analyzed annual grain yield data for rice, wheat, and maize that were extracted from the China Statistical Yearbook. In addition, to establish a comprehensive dataset on long-term changes in the nutrient composition of rice, wheat, and maize, the Web of Science, Google Scholar, and China National Knowledge Infrastructure (CNKI) databases were searched for relevant studies published between January 1980 and December 2024. Combinations of crop-related keywords, including “rice” or “wheat” or “maize” or “corn” or “cereal” or “grain,” and nutrient-related keywords, including “protein” or “fat” or “lipid” or “carbohydrate” or “starch” or “moisture” or “dietary fiber” or “nutrient composition” or “nutritional quality” or “grain quality” or “proximate composition,” were used. The corresponding Chinese terms were used as keywords in CNKI. The same keyword combinations were used across databases, and the search fields and syntax were adjusted on the basis of the requirements of each database.

**Figure 1:**
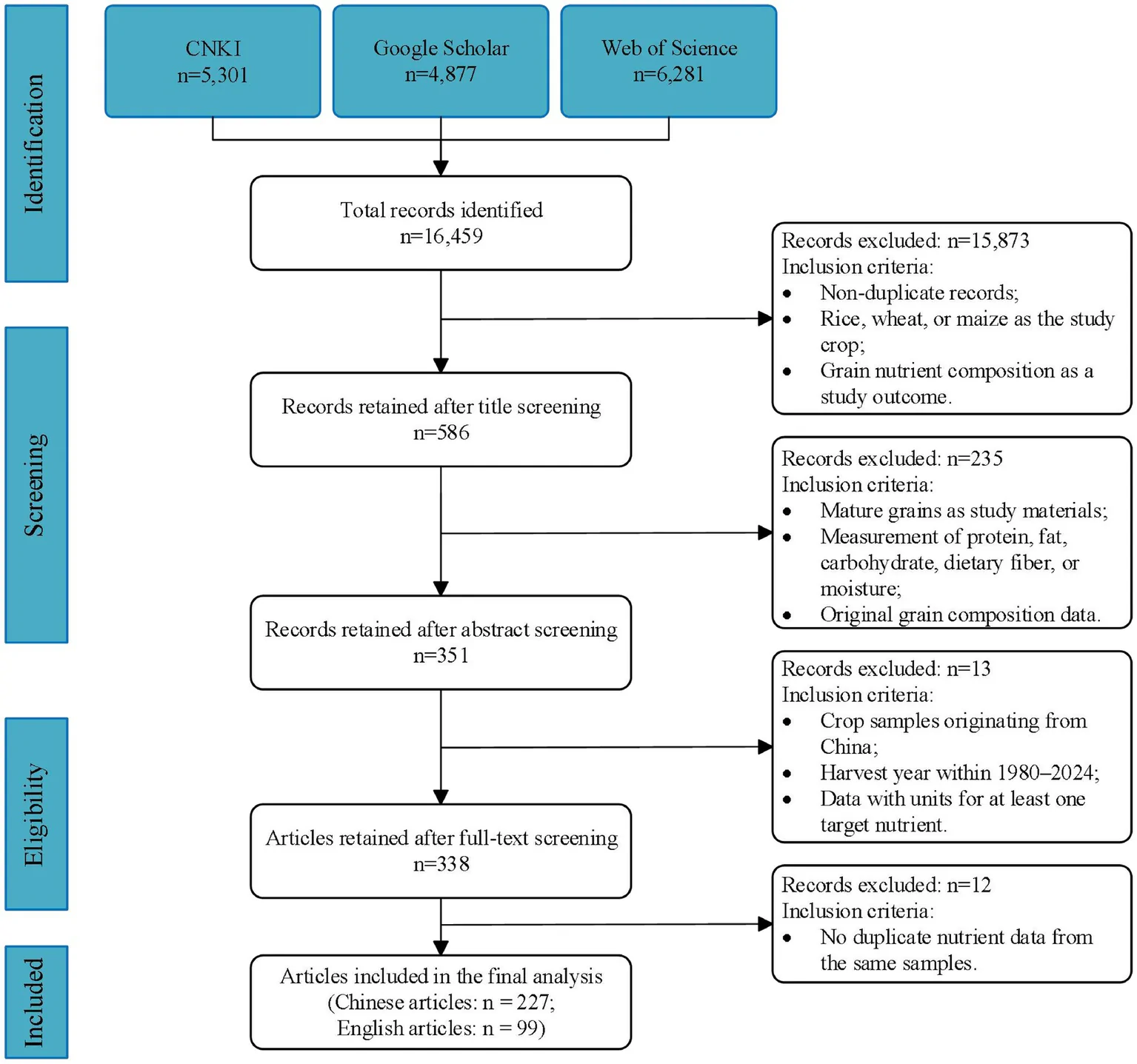
Flow diagram of literature screening process.

Literature screening was independently performed by multiple researchers according to predefined criteria. Both abstract screening and full-text evaluation were independently performed by two researchers. The initial search retrieved a total of 16,459 records: 5,301 from CNKI, 4,877 from Google Scholar, and 6,281 from Web of Science. Duplicates were removed, and the titles and abstracts of the remaining records were screened using the following inclusion criteria: (a) samples consisted of mature grain (excluding silage wheat, silage maize, fresh sweet maize, and waxy maize), (b) samples originated from China, (c) the publication reported a clearly defined harvest year, and (d) data for at least one nutrient composition indicator were provided. In total, 15,873 records were excluded because they failed to meet one or more of the criteria. Studies were most commonly excluded because they were conducted outside China, did not report a harvest year, lacked nutrient composition data, examined processed grain products, or involved controlled experiments under nonnatural production conditions. A total of 586 records underwent full-text evaluation. Of these, 235 were excluded on the basis of an abstract review because they were determined to be unrelated to the research topic, 13 were excluded following full-text review because they failed to meet the inclusion criteria, and 12 were excluded because of incomplete data. Finally, 326 records were included for data extraction and analysis. The complete screening process is presented in Figure 1. The resulting nutrient composition data for rice, wheat, and maize grains are summarized in Table 1.

**Table 1:** Descriptive statistics of nutrient composition of rice, wheat, and maize grains.

Crop	Nutrient component	*N*	Content (g/100 g)			
			Minimum	Maximum	Mean	SD
Rice	Protein	1,253	4.600	15.410	9.137	1.839
	Fat	227	0.200	4.380	1.612	1.014
	Carbohydrates	284	52.610	89.400	75.098	7.326
	Moisture	195	5.810	17.230	12.495	2.143
	Fiber	40	0.170	5.190	2.057	1.754
Wheat	Protein	1834	9.800	18.910	13.832	1.640
	Fat	153	0.900	3.340	1.925	0.548
	Carbohydrates	538	48.070	81.100	66.054	7.052
	Moisture	141	8.200	15.700	12.129	1.706
	Fiber	60	0.100	14.400	9.697	4.609
Maize	Protein	228	2.600	14.670	9.025	1.776
	Fat	202	2.283	11.190	4.098	1.019
	Carbohydrates	150	51.000	77.760	71.326	4.488
	Moisture	55	8.810	15.450	11.417	1.624
	Fiber	65	0.770	13.170	4.128	2.958

SD, standard deviation.

### 2.2 Data analysis

Statistical analyses were conducted using Stata (version 2018; StataCorp, College Station, TX, USA). Trend plots were generated using Origin (version 2021; OriginLab Corporation, Northampton, MA, USA) to visualize temporal changes in the grain yield and nutrient composition of rice, wheat, and maize.

For each crop, the China Statistical Yearbook reports a national mean grain yield per unit area for each year. Therefore, linear regression models were fitted for rice, wheat, and maize to assess the relationship between annual grain yield per unit area and year. The model was specified as follows:


$$Y_{i}=\beta_{0}+\beta_{1}X_{i}+\mu_{i}$$

Here, *Y_i_* denotes the grain yield per unit area (t/ha) in year *i*, *X_i_* represents the corresponding harvest year, *β_0_* is the intercept, *β_1_* represents the average annual change in grain yield per unit area, and *μ_i_* is the random error term.

Regarding the grain nutrient composition data, multiple observations were sometimes available for the same cultivar and harvest year. To avoid assigning disproportionate weight to repeatedly reported cultivar–year combinations, observations were first averaged within each cultivar–year combination; this yielded one mean value per cultivar per year. Subsequently, separate linear regression models were fitted for each crop and nutrient variable to assess the association between nutrient concentration and harvest year. The model was specified as follows:


$$W_{\mathrm{ij}}=\beta_{0}+\beta_{1}X_{i}+\mu_{\mathrm{ij}}$$

Here, *W_ij_* denotes the mean concentration (g/100 g) of the nutrient (protein, fat, carbohydrates, dietary fiber, or moisture) in cultivar *j* harvested in year *i*, *X_i_* denotes the corresponding harvest year, *β_0_* is the intercept, *β_1_* represents the average annual change in nutrient concentration, and *μ_ij_* is the random error term.

To evaluate the relationship between grain yield and nutrient composition, *t* each nutrient concentration observation was matched with the corresponding national grain yield per unit area (t/ha) for the same crop and harvest year. Separate simple linear regression models were then fitted for each crop and nutrient variable to assess the association between grain yield and nutrient concentration. The model was specified as follows:


$$W_{\mathrm{ij}}=\beta_{0}+\beta_{1}Y_{i}+\mu_{\mathrm{ij}}$$

Here, *W_ij_* denotes the mean concentration (g/100 g) of the nutrient in cultivar *j* harvested in year *i*, *Y_i_* denotes the corresponding national mean grain yield per unit area (t/ha) in year *i*, *β0* is the intercept, *β1* represents the estimated change in nutrient concentration associated with a 1 t/ha increase in grain yield, and *μ_ij_* is the random error term.

## 3 Results

### 3.1 Trends in grain yield

**Figure 2:**
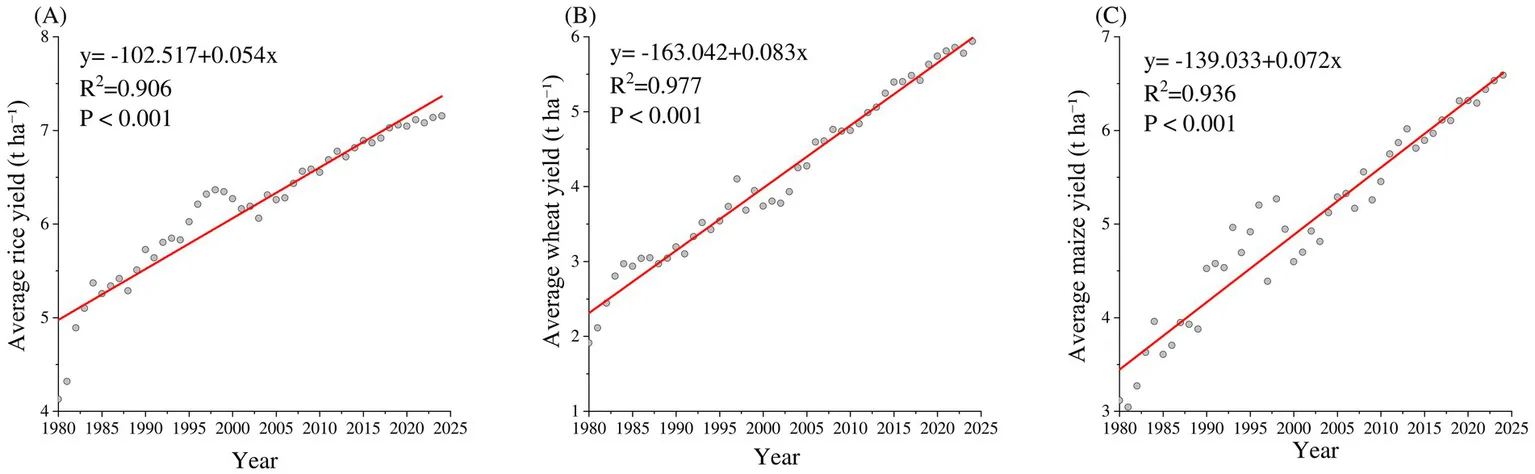
Linear trends in yield per unit area of rice, wheat, and maize from 1980 to 2024. **(A)** Rice; **(B)** Wheat; **(C)** Maize.

Figure 2 presents the temporal trends in grain yield of rice, wheat, and maize from 1980 to 2024. Overall, grain yields of all three crops showed sustained increases over the study period, although the magnitude and pace of improvement differed among crops. Wheat exhibited the largest annual increase, with yield increasing by 0.083 t ha^−1^ year^−1^ (R^2^ = 0.977, *p* < 0.001), followed by maize (0.072 t ha^−1^ year^−1^, R^2^ = 0.936, *p* < 0.001) and rice (0.054 t ha^−1^ year^−1^, R^2^ = 0.906, *p* < 0.001).

This pattern was further reflected in the period-specific means presented in Supplementary Table S1. The mean yield across the three crops increased from 3.539 to 6.455 t ha^−1^ from 1980 to 2024, representing an increase of 82.4%. Among the three crops, wheat exhibited the largest proportional increase; the grain yield increased from 2.450 t ha^−1^ in 1980–1984 to 5.826 t ha^−1^ in 2020–2024, representing an approximately 137.8% increase. This was followed by maize, which increased from 3.404 t ha^−1^ to 6.434 t ha^−1^, representing an approximately 89.0% increase. Rice showed the smallest proportional increase, increasing by approximately 49.2% from 4.762 t ha^−1^ to 7.106 t ha^−1^. Notably, both rice and maize exhibited a slight decline around 2000–2004.

### 3.2 Trends in major nutrient contents

#### 3.2.1 Protein

**Figure 3:**
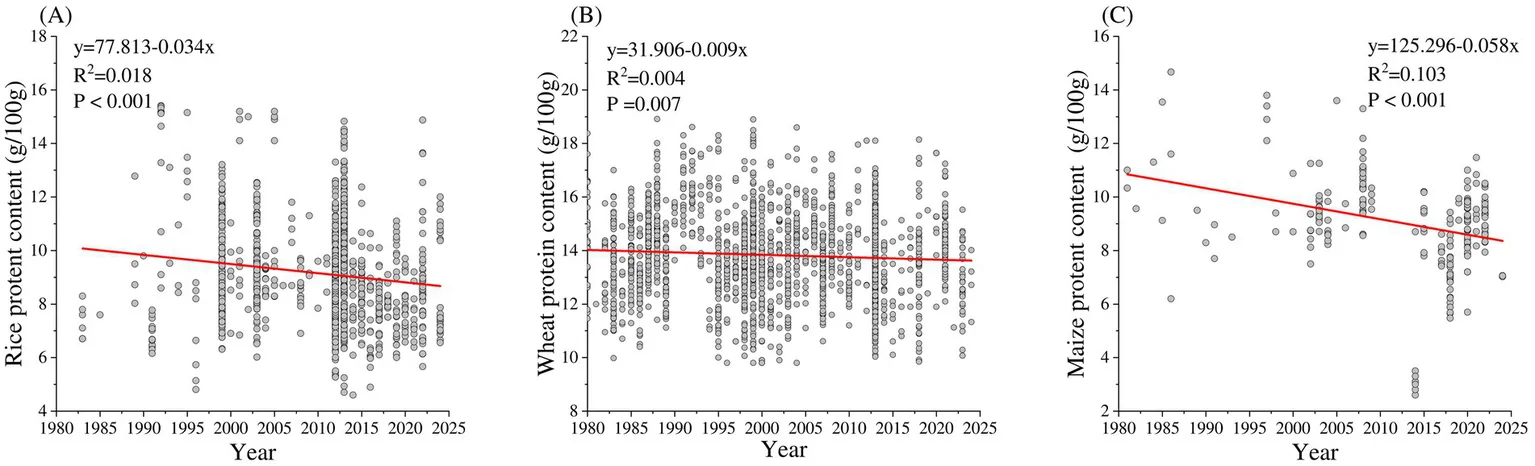
Linear trends in protein concentrations in rice, wheat, and maize from 1980 to 2024. **(A)** Rice; **(B)** Wheat; **(C)** Maize.

Linear regression analyses revealed that the protein concentrations in the three major grain crops significantly declined over the study period (Figure 3), with estimated average annual decreases of 0.034 g/100 g year^−1^ in rice (R^2^ = 0.018, *p* < 0.001), 0.009 g/100 g year^−1^ in wheat (R^2^ = 0.004, *p* = 0.007), and 0.058 g/100 g year^−1^ in maize (R^2^ = 0.103, *p* < 0.001). Although harvest year accounted for only a limited proportion of the variation in grain protein concentration, significant declining trends were consistently observed across all three crops.

The period-specific means provided a more detailed picture of these changes (Supplementary Table S2). Among the three crops, wheat grain maintained the highest protein concentration throughout the study period. The protein concentration in wheat grain steadily increased from 13.469 g/100 g in 1980–1984 to a peak of 15.595 g/100 g in 1990–1994. It then declined to 13.927 g/100 g in 2020–2024, representing a reduction of approximately 10.70% relative to the peak value. The protein concentration in rice grain followed a broadly similar pattern, rising from 7.367 g/100 g in 1980–1984 to a peak of 10.139 g/100 g in 2005–2009, then declining to 8.478 g/100 g in 2020–2024. The protein concentration in maize grain fluctuated considerably between 1980 and 2009, generally remaining around 10 g/100 g during this period. After 2010, it declined further and consistently remained below 10 g/100 g, reaching 9.086 g/100 g in 2020–2024.

#### 3.2.2 Fat

**Figure 4:**
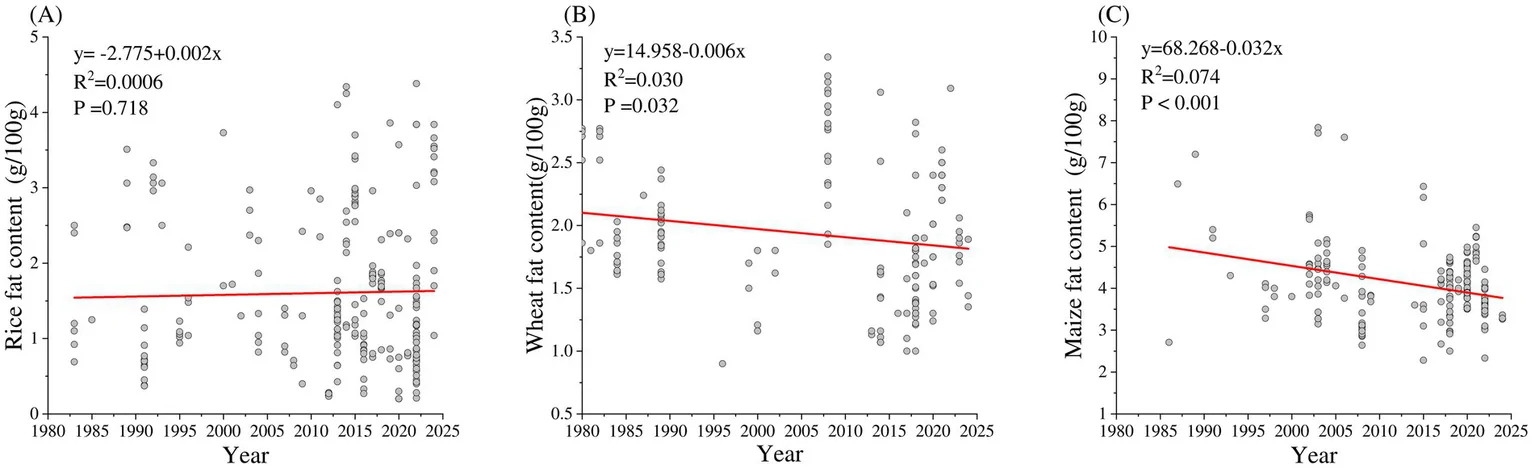
Linear trends in fat concentrations in rice, wheat, and maize from 1980 to 2024. **(A)** Rice; **(B)** Wheat; **(C)** Maize.

Linear regression analysis revealed significant downward trends in fat concentrations in wheat (−0.006 g/100 g year^−1^, R^2^ = 0.030, *p* = 0.032) and maize (−0.032 g/100 g year^−1^, R^2^ = 0.074, *p* < 0.001), whereas no clear temporal trend was noted in rice (Figure 4). Despite the limited explanatory power of harvest year for grain fat concentration, significant declining trends were detected in wheat and maize over the study period.

The temporal variation in mean fat concentrations among the three crops was further summarized (Supplementary Table S3). Overall, the mean fat concentration across the three crops increased from 1.801 g/100 g in 1980–1984 to 3.325 g/100 g in 1985–1989. It then declined with fluctuations and stabilized within a range of approximately 2.147 to 2.746 g/100 g, reaching 2.520 g/100 g in 2020–2024. The fat concentration in rice grain fluctuated considerably between 1980 and 2024, with no clear temporal trend observed. The mean concentration ranged from 1.100 to 2.554 g/100 g across the study period. The fat concentration in wheat grain initially increased and then decreased. It increased from 2.133 g/100 g in 1980–1984 to a peak of 2.686 g/100 g in 2005–2009. Starting in 2010, it markedly decreased, reaching 1.944 g/100 g in 2020–2024. The fat concentration in maize grain generally decreased. It declined from 5.467 g/100 g in 1985–1989 to 3.600 g/100 g in 2010–2014. It then recovered slightly to 4.002 g/100 g in 2020–2024.

#### 3.2.3 Carbohydrates

**Figure 5:**
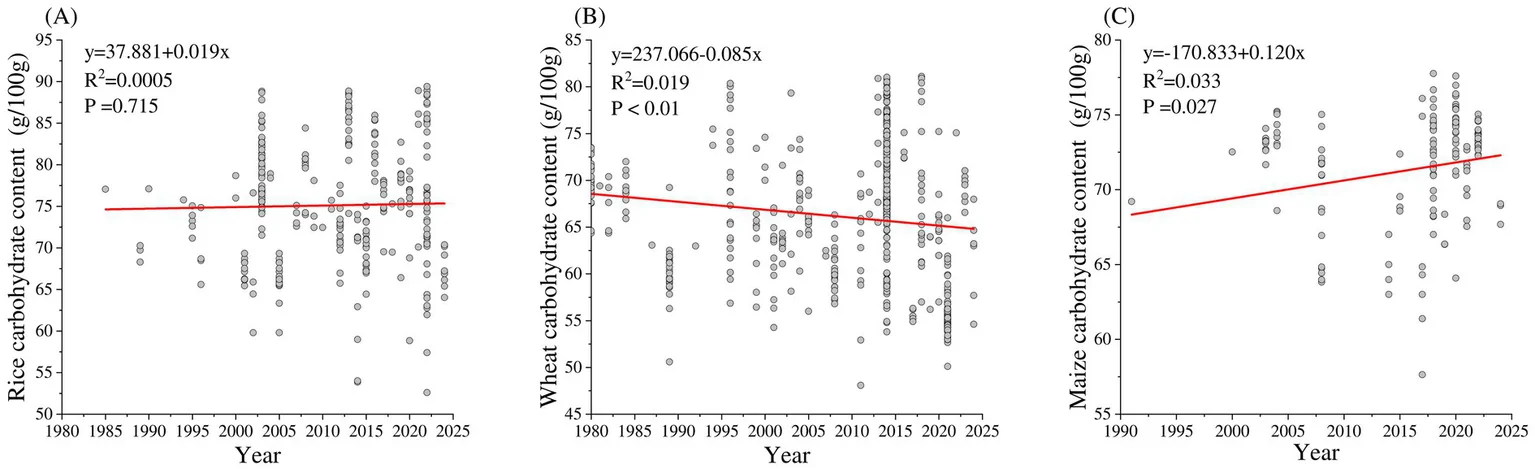
Linear trends in carbohydrate concentrations in rice, wheat, and maize from 1980 to 2024. **(A)** Rice; **(B)** Wheat; **(C)** Maize.

Linear regression analysis revealed a downward trend in carbohydrate concentration in wheat (0.085 g/100 g year^−1^, R^2^ = 0.019, *p* = 0.001) and an upward trend in carbohydrate concentration in maize (0.120 g/100 g year^−1^, R^2^ = 0.033, *p* = 0.027); however, no clear temporal trend was noted in rice (Figure 5). Although harvest year accounted for only a limited proportion of the variation in grain carbohydrate concentration, the downward trend in wheat and the upward trend in maize were both statistically significant.

The mean carbohydrate concentration across the three crops fluctuated from 65.762 to 72.121 g/100 g across the study period, with no consistent long-term direction (Supplementary Table S4). Among the three crops, rice had the highest carbohydrate concentration. Its carbohydrate concentration remained within a range of 71–77 g/100 g, reaching a peak of 76.799 g/100 g in 2000–2004. Thereafter, it generally decreased, reaching 74.427 g/100 g in 2020–2024. The carbohydrate concentration in wheat decreased from 69.251 g/100 g in 1980–1984 to 58.750 g/100 g in 2020–2024, representing a cumulative decrease of approximately 15.2%. The carbohydrate concentration in maize fluctuated substantially over time. It increased from 69.200 g/100 g in 1990–1994 to 72.216 g/100 g in 2000–2004, decreased to 60.500 g/100 g in 2010–2014, and then gradually increased in 2015–2024, reaching 73.036 g/100 g in 2020–2024.

#### 3.2.4 Fiber and moisture

**Figure 6:**
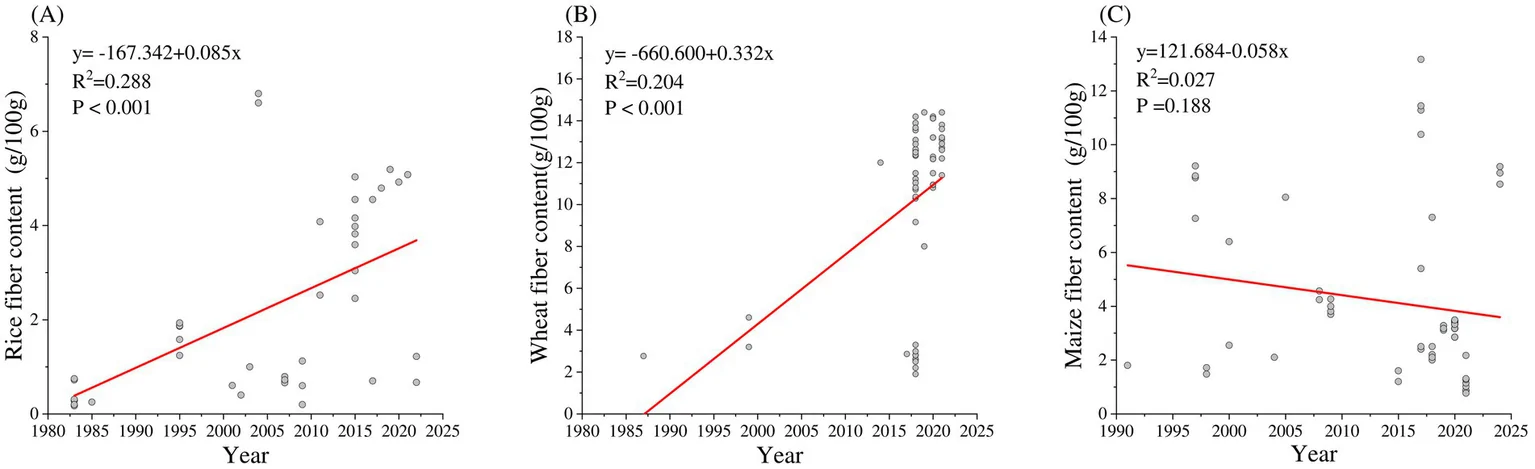
Linear trends in dietary fiber contents in rice, wheat, and maize from 1980 to 2024. **(A)** Rice; **(B)** Wheat; **(C)** Maize.

Linear regression analysis revealed upward trends in the dietary fiber contents in rice (0.085 g/100 g year^−1^, R^2^ = 0.288, *p* < 0.001) and wheat (0.332 g/100 g year^−1^, R^2^ = 0.204, *p* < 0.001); however, no clear temporal trend was noted in maize (Figure 6). Although variation in dietary fiber concentration was not fully explained by harvest year, significant increases were observed in rice and wheat.

The mean dietary fiber content across the three crops increased from 0.405 g/100 g in 1980–1984 to 6.325 g/100 g in 2020–2024 (Supplementary Table S5). Among the three crops, wheat exhibited the highest dietary fiber content throughout most of the study period, increased from 2.760 g/100 g in 1985–1989 to 12.706 g/100 g in 2020–2024. The dietary fiber content in rice generally increased, despite some fluctuations. It increased from 0.405 g/100 g in 1980–1984 to 3.821 g/100 g in 2015–2019. Although it decreased to 2.973 g/100 g in 2020–2024, it remained higher than the levels observed before 2000. The dietary fiber content in maize initially increased and then decreased. It increased from 1.800 g/100 g in 1990–1994 to 6.213 g/100 g in 1995–1999. Subsequently, it decreased and fluctuated within a range of 3.297–4.813 g/100 g between 2000 and 2024, consistent with the absence of a significant temporal trend.

**Figure 7:**
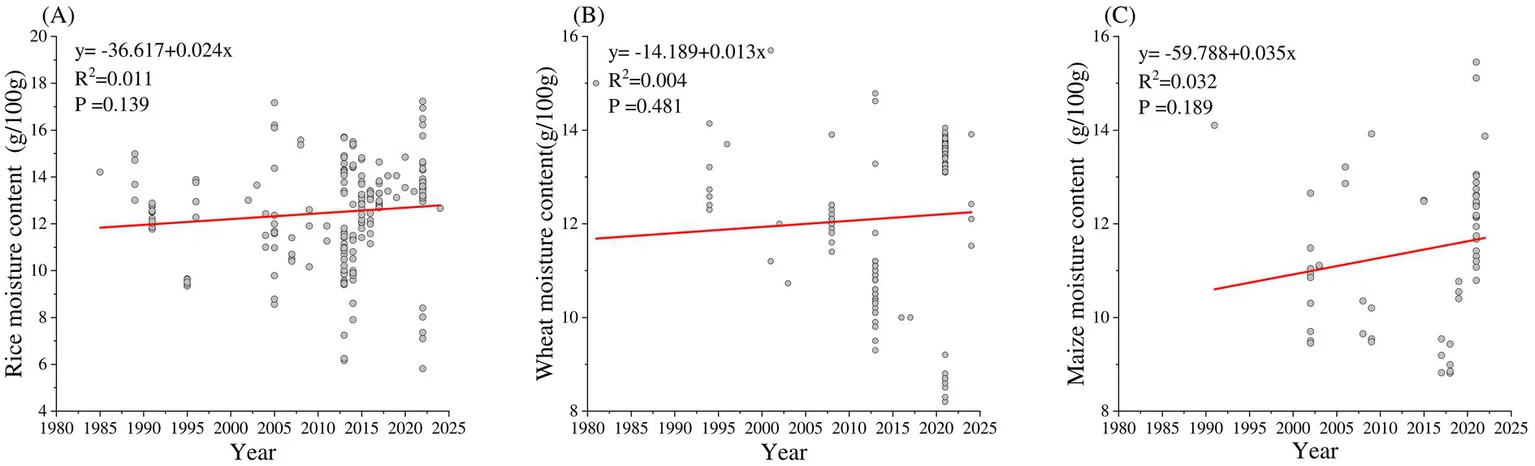
Linear trends in moisture contents in rice, wheat, and maize from 1980 to 2024. **(A)** Rice; **(B)** Wheat; **(C)** Maize.

Linear regression analysis revealed that the moisture contents of rice, wheat, and maize remained largely stable throughout 1980–2024, with no clear temporal trends noted for any of the three crops (Figure 7). The period-specific mean values summarized provide further insight into the temporal variation in moisture content (Supplementary Table S6). The overall mean moisture content across the three crops decreased from 15.000 g/100 g during 1980–1984 to 11.013 g/100 g during 2015–2019, followed by an increase to 12.786 g/100 g during 2020–2024. At the crop level, the moisture contents in rice, wheat, and maize initially decreased and then increase. In rice, the moisture content decreased from 14.114 g/100 g in 1985–1989 to 11.155 g/100 g in 1995–1999. It subsequently fluctuated within a range of 11–13 g/100 g before increasing to 13.173 g/100 g in 2020–2024. In wheat, the moisture content decreased from 15.000 g/100 g in 1980–1984 to 10.000 g/100 g in 2015–2019, which was the lowest level observed during the study period. It then increased to 12.743 g/100 g in 2020–2024. Additionally, in maize, the moisture content generally decreased from 14.100 g/100 g in 1990–1994 to 10.028 g/100 g in 2015–2019 before recovering to 12.443 g/100 g in 2020–2024.

### 3.3 Relationship between grain yield and nutrient contents

Linear regression analysis revealed negative relationships between grain protein concentration and grain yield in rice (−0.948, R^2^ = 0.029, *p* < 0.001), wheat (−0.102, R^2^ = 0.003, *p* < 0.05), and maize (−0.747, R^2^ = 0.099, *p* < 0.001) (Table 2). Although grain yield explained only a small proportion of the variation in grain protein concentration, the significant negative relationships indicated that higher grain yields were associated with lower protein concentrations across the three crops. Fat concentration also showed negative relationships with grain yield in wheat (−0.075, R^2^ = 0.028, *p* = < 0.05) and maize (−0.464, R^2^ = 0.088, *p* < 0.001), whereas no significant relationship was detected in rice.

**Table 2:** Linear regression relationships between grain yield and nutrient concentrations.

Nutrient component	Statistic	Rice	Wheat	Maize
Protein	Yield	−0.948***	−0.102*	−0.747***
	Constant	15.442	14.246	13.315
	R^2^	0.029	0.003	0.099
	*N*	1,255	1834	228
Fat	Yield	0.052	−0.075*	−0.464***
	Constant	1.258	2.268	6.802
	R^2^	0.001	0.028	0.088
	*N*	229	153	202
Carbohydrate	Yield	−0.470	−0.895**	1.472*
	Constant	78.237	70.341	62.517
	R^2^	0.001	0.017	0.027
	*N*	286	538	150
Dietary fiber	Yield	1.678***	4.373***	−0.955
	Constant	−8.396	−13.735	9.779
	R^2^	0.31	0.245	0.041
	*N*	42	57	65
Moisture	Yield	0.449	0.166	0.505
	Constant	9.49	11.255	8.484
	R^2^	0.008	0.005	0.034
	*N*	197	141	55

Values represent regression coefficients. Significance levels are indicated as ***p < 0.001, **p < 0.01, and *p < 0.05.

In contrast, linear regression analysis revealed positive relationships between dietary fiber concentration and grain yield in rice (1.678, R^2^ = 0.310, *p* < 0.001) and wheat (4.373, R^2^ = 0.245, *p* < 0.001). These positive relationships indicated that higher grain yields were associated with higher dietary fiber concentrations in these two crops, whereas no significant relationship was detected in maize. The relationships between yield and carbohydrate concentration differed among crops. Carbohydrate concentration showed no significant relationship with yield in rice, a negative relationship was observed in wheat (−0.895, R^2^ = 0.017, *p* < 0.01), and a positive relationship was observed in maize (1.472, R^2^ = 0.027, *p* < 0.05). No significant relationship was detected between grain yield and moisture concentration in any of the three crops.

## 4 Discussion

### 4.1 Overview of yield and nutrient trends

Our results revealed that grain yields of rice, wheat, and maize in China increased overall between 1980 and 2024, although the rate and timing of yield improvement differed among crops. Accompanying these increases in grain yield, changes in grain nutrient composition occurred over time and exhibited distinct patterns among nutrients and crops. Protein exhibited a consistent decline across the three cereals, with significant decreases in rice, wheat, and maize and negative associations between yield and protein concentration. Fat concentrations declined in wheat and maize but remained unchanged in rice, whereas carbohydrate concentrations decreased in wheat, increased in maize, and showed no significant change in rice. Dietary fiber increased in rice and wheat but not in maize, while moisture content showed relatively limited long-term variation.

Together, these results demonstrate that long-term changes in grain nutrient composition were not uniform but varied substantially among nutrient components and crops. These diverse patterns likely reflect the combined influences of genetic improvement, agricultural management practices, and environmental changes occurring alongside long-term increases in crop productivity over the past four decades. These factors may influence grain nutrient composition through two complementary mechanisms: nutrient dilution associated with increased biomass accumulation and yield improvement, and direct changes in grain composition associated with genetic improvement, management practices, and environmental conditions.

### 4.2 Drivers of the long-term increase in grain yield

Genetic improvement, agricultural management, and climate change have each contributed to the long-term increase in grain yield observed in this study, although their relative contributions have varied over time. Five-year period averages indicate that grain yield in rice and maize was slightly lower during 2000–2004 than that during 1995–1999, whereas wheat yield increased only marginally over the same period. Yield growth subsequently recovered from 2005 to 2009 onward across all three crops. This temporary slowdown coincided with structural adjustments in China’s grain sector during the early 2000s. Following the decline in grain prices after the late 1990s, grain production decreased from 512 to 431 million tons between 1998 and 2003 (13). Subsequent policy interventions, including the abolition of agricultural taxes, direct grain subsidies, and minimum purchase price policies, supported the recovery of grain yield improvement (14).

Beyond these short-term fluctuations, the overall yield increase across the full study period was largely driven by the combined effects of genetic improvement, agricultural intensification, and climate change. Genetic improvement has played a fundamental role in increasing yield potential through the selection and adoption of cultivars with improved plant architecture, lodging resistance, resource-use efficiency, and adaptation to intensive production systems. For rice and wheat, the introduction of semi-dwarfing alleles, such as sd1 in rice and Rht alleles in wheat, enhanced lodging resistance and improved yield performance under intensive production systems (15–17). In maize, yield improvement has mainly resulted from advances in hybrid breeding, heterosis utilization, improved plant architecture, and increased tolerance to high planting densities, allowing modern hybrids to maintain productivity under intensive management systems (18).

Agricultural intensification has further supported yield gains through increased fertilizer application, expanded irrigation coverage, higher planting density, improved crop management practices, and mechanization. Globally, yield is strongly determined by nutrient and water management, and closing yield gaps through improved fertilizer and irrigation practices could increase production of major cereals by approximately 45–70% (19). In China, rapid increases in fertilizer inputs and agricultural infrastructure since the late twentieth century have contributed substantially to cereal productivity growth, although the marginal benefits of further input increases have declined with increasing application intensity (20).

Environmental changes, particularly rising atmospheric CO_2_ concentrations, may have provided an additional contribution to long-term yield increases. Elevated CO_2_ can stimulate photosynthetic carbon assimilation, biomass accumulation, and grain yield in C₃ crops such as rice and wheat through the CO_2_ fertilization effect. A meta-analysis of two decades of free-air CO_2_ enrichment experiments showed that elevated CO₂ increased rice yield by approximately 16.2% on average, with stronger responses observed under specific cultivar and nutrient conditions (21). Collectively, genetic advances, improved agricultural management, and environmental changes represent interconnected drivers of China’s long-term grain yield increases. However, the same processes that enhanced crop productivity may also have affected grain nutrient composition through multiple pathways, including nutrient dilution associated with increased biomass production and crop-specific changes arising from breeding objectives, management practices, and environmental conditions, as discussed below.

### 4.3 Drivers of long-term changes in grain nutrient composition

In the present study, grain yield was negatively associated with protein concentration in rice, wheat, and maize. One possible explanation for this pattern is the nutrient dilution effect, which occurs when biomass production increases more rapidly than nutrient accumulation, thereby reducing nutrient concentrations (22, 23). This phenomenon can occur under different yield-improving conditions, including genetic improvement, agricultural management, and environmental changes, when increases in biomass production exceed the corresponding increases in nutrient accumulation.

Dilution associated with genetic improvement has been observed in long-term cultivar comparisons. Consistent with this pattern, a long-term analysis of U. S. wheat cultivars released between 1870 and 2013 showed that release year was negatively associated with grain protein concentration (24). Evidence of declines in other grain nutrients has also been reported. A century-scale field experiment in the United Kingdom showed that concentrations of several grain nutrients declined after the introduction of semi-dwarf, high-yielding wheat cultivars in the 1960s (25). Similar trends have been reported in Chinese maize cultivars released between 1930 and 2010, with yield increases accompanied by declines in multiple grain nutrients (26). Long-term declines in grain mineral density have also been reported for widely adopted high-yielding rice and wheat cultivars released in India between the 1960s and the 2010s (27).

Beyond dilution, changes in breeding priorities can independently reshape grain composition. For rice, selection for improved eating quality may have lowered grain protein concentration, as higher protein is generally associated with poorer eating and cooking quality (28). Among japonica rice cultivars released in the Yangtze River Basin from the 1950s to the 2000s, improved eating and cooking quality was accompanied by lower grain protein content (29). Breeding for eating quality may also be associated with changes in starch-related traits. In this study, no significant temporal trend was detected in total carbohydrate concentration in rice, whereas Feng et al. (9) reported a decrease in amylose concentration among indica hybrid rice cultivars released between 2000 and 2014. These findings are not contradictory because amylose is only one component of starch. Wheat breeding has likewise emphasized end-use quality. In northern China, where steamed bread and Chinese noodles are the main wheat products, improving dough strength has been an important breeding objective because both products generally require moderate dough strength (30). In this study, maize carbohydrate concentration increased significantly over time. Consistent with this finding, a comparison of Chinese maize hybrids released from the 1960s to the 2000s showed that starch concentration increased by 0.81% per decade. Relative to U. S. hybrids released over the same period, Chinese hybrids had lower starch and higher protein concentrations overall, highlighting differences in breeding priorities between the two countries (31). Maize breeding priorities have been shaped by demand from the feed, starch-processing, and biofuel industries (32); bioethanol pilot programs increased processing-oriented maize demand in the early 2000s, before food-security concerns prompted restrictions on grain-based ethanol from 2007, with support later renewed from 2016 to help reduce maize stockpiles (33). Selection for higher starch concentration may thus have contributed to the observed increase in maize carbohydrate concentration.

Agricultural management can contribute to nutrient dilution by altering the balance between biomass production and nutrient accumulation. A global synthesis of 243 field studies across 41 countries reported consistent declines in wheat grain zinc, iron, and protein concentrations since the 1960s, coinciding with agricultural intensification (34). Fertilizer management is particularly important because it can alter nutrient availability, nitrogen use efficiency, dry matter accumulation, and the relative allocation of carbon and nitrogen to grain protein and carbohydrates (35, 36). Evidence from rice further indicates that grain yield and protein concentration are closely linked through shared nitrogen pathways and are both strongly influenced by nitrogen management (37). Balanced fertilizer management can increase yield and grain protein concentration under certain conditions (38), whereas excessive or imbalanced fertilizer application may disrupt nutrient acquisition and reduce the concentrations of some nutrients (39, 40). Notably, grain protein concentrations in all three crops reached their lowest levels during 2015–2019 in China, coinciding with the historical peak in fertilizer consumption in 2015 and subsequent implementation of the Action Plan for Zero Growth in Chemical Fertilizer Use by 2020 (41). This temporal association may also reflect concurrent changes in cultivars, climate, and other management practices.

Rising atmospheric CO_2_ can stimulate photosynthesis and carbohydrate accumulation in C_3_ crops such as rice and wheat, diluting grain protein concentration as carbon and biomass accumulation outpace nitrogen acquisition, while also potentially contributing to this decline by altering nitrogen uptake, assimilation, and remobilization. A large synthesis of free-air CO_2_ enrichment experiments found that elevated CO_2_ reduced zinc, iron, and protein concentrations in rice and wheat grains, whereas maize exhibited comparatively smaller changes (42–44); consistent with this broader pattern, studies focusing specifically on rice and wheat have reported approximately 7–15% reductions in grain protein concentrations under elevated CO_2_ conditions (45, 46). A systematic review similarly reported reductions in iron and zinc concentrations in rice grains grown under elevated CO_2_ (47). However, evidence from long-term field observations under gradual increases in atmospheric CO_2_ is not entirely consistent with results from experimental CO_2_ enrichment studies. Bloom and Plant (2021) analyzed wheat field trials conducted in California from 1985 to 2019, during which atmospheric CO_2_ concentration increased by 19%. After adjustment for major agronomic and environmental factors, grain yield and grain protein yield declined by 13%, whereas grain protein concentration remained unchanged, possibly due to compensatory adjustments in carbon fixation and nitrate assimilation under gradual CO₂ enrichment (48).

Together, these findings suggest that the observed long-term changes in grain nutrient composition were shaped by multiple interacting processes. Nutrient dilution provides a common mechanism linking increasing productivity with declining concentrations of some nutrients, whereas breeding objectives, management practices, and climate factors may additionally contribute to crop-specific changes in grain composition through other pathways.

### 4.4 Strengths and limitations

This study has several strengths. First, it systematically compiles a long-term database covering the yield and nutrient composition of China’s three major staple crops over more than four decades (1980–2024), providing an empirical foundation for future research on long-term trends in grain nutrient composition and their underlying drivers. Second, prior research on long-term changes in grain nutrient composition has focused predominantly on Europe and North America (e.g., U. S. maize, European wheat), leaving systematic evidence on China’s staple crops comparatively scarce; this study provides a systematic characterization of long-term nutrient trends in Chinese rice, wheat, and maize. Third, whereas most existing studies have examined a single crop or a limited set of nutrients, this study jointly analyzes long-term trends in protein, fat, carbohydrate, and dietary fiber across three staple crops, broadening the scope of existing research and offering a more comprehensive perspective on the evolution of grain nutritional quality in China.

Nevertheless, this study also has several limitations. First, due to the complexity and availability of long-term grain nutrient composition data, this study focused on quantifying temporal trends in grain yield and nutrient composition. The potential effects of genetic improvement, agricultural management, and environmental change were discussed based on previous studies and theoretical mechanisms; however, their relative contributions could not be quantitatively evaluated. Second, this study mainly examined the overall trends in grain yield and nutrient composition at the national scale, and did not further investigate regional variations or differences among cropping systems. Future research should integrate more detailed datasets to identify the factors driving changes in the grain yield and nutrient composition of major staple crops from multiple perspectives. First, climate variables, including temperature, precipitation, and atmospheric CO₂ concentration, together with agricultural input records such as fertilizer application, irrigation, and soil properties, could be integrated with grain yield and nutrient composition datasets and analyzed using panel-data or multilevel models to quantify the contributions of environmental and management factors. Second, future studies could incorporate regional-scale datasets to investigate spatial heterogeneity in grain yield and nutrient composition trends across different regions and agroecological zones. Such analyses would help identify regional differences in long-term changes and provide a more comprehensive understanding of variations among different production areas.

## 5 Conclusion

This study systematically analyzed the long-term trends in the grain yield and nutrient composition of three major staple crops in China between 1980 and 2024. The findings revealed that the nutrient composition of these crops substantially changed over this period. The changes involve a decrease in grain protein concentrations and an increase in dietary fiber concentrations. From a policy perspective, efforts to safeguard food security should place greater emphasis on nutritional quality. Nutritional quality traits should be incorporated into breeding objectives and cultivar approval criteria to support the development of high-yielding cultivars with favorable nutrient profiles. Fertilizer management should also be improved to enhance nitrogen use efficiency. Moreover, a national monitoring system for the nutrient composition of staple crops should be established to inform dietary guidelines, nutrition policies, and targeted public health interventions. These measures could jointly support improvements in the dietary nutritional quality of the population alongside food security goals.

## Data Availability

The raw data supporting the conclusions of this article will be made available by the authors, without undue reservation.
