# Supplementary material for: Long-term trends in grain yield and main nutrient contents of three staple cereal crops in China (1980–2024)
Source: Front Nutr. 2026 Sep 7;13:1936187. doi: 10.3389/fnut.2026.1936187 (PMC13590387; doi:10.3389/fnut.2026.1936187)
Supplement: Supplementary file 1 [file Table_1.DOCX]

Supplementary Material

TABLE S1 Changes in grain yield of staple crops in China across 5-year periods from 1980 to 2024.

| Period | Mean grain yield (t ha^−1^) | | | |
| --- | --- | --- | --- | --- |
|  | Mean | Rice | Wheat | Maize |
| 1980–1984 | 3.539 | 4.762 | 2.450 | 3.404 |
| 1985–1989 | 4.061 | 5.361 | 3.007 | 3.814 |
| 1990–1994 | 4.581 | 5.77 | 3.314 | 4.658 |
| 1995–1999 | 5.000 | 6.253 | 3.802 | 4.944 |
| 2000–2004 | 4.977 | 6.199 | 3.901 | 4.831 |
| 2005–2009 | 5.446 | 6.424 | 4.596 | 5.319 |
| 2010–2014 | 5.822 | 6.710 | 4.976 | 5.779 |
| 2015–2019 | 6.165 | 6.952 | 5.465 | 6.078 |
| 2020–2024 | 6.455 | 7.106 | 5.826 | 6.434 |

TABLE S2 Changes in grain protein concentrations in the three major staple crops in China from 1980 to 2024.

| Period | Grain protein concentration (g/100 g) | | | |
| --- | --- | --- | --- | --- |
|  | Mean | Rice | Wheat | Maize |
| 1980–1984 | 10.461 | 7.367 | 13.469 | 10.548 |
| 1985–1989 | 11.382 | 9.326 | 14.049 | 10.772 |
| 1990–1994 | 11.295 | 9.926 | 15.595 | 8.363 |
| 1995–1999 | 11.566 | 9.343 | 13.637 | 11.717 |
| 2000–2004 | 10.788 | 9.549 | 13.552 | 9.263 |
| 2005–2009 | 11.540 | 10.139 | 14.114 | 10.366 |
| 2010–2014 | 10.585 | 9.242 | 13.657 | 8.857 |
| 2015–2019 | 9.727 | 8.140 | 13.165 | 7.875 |
| 2020–2024 | 10.497 | 8.478 | 13.927 | 9.086 |

TABLE S3 Changes in fat concentrations in the three major staple crops in China from 1980 to 2024.

| Period | Grain fat concentration (g/100 g) | | | |
| --- | --- | --- | --- | --- |
|  | Mean | Rice | Wheat | Maize |
| 1980–1984 | 1.801 | 1.468 | 2.133 | - |
| 1985–1989 | 3.325 | 2.554 | 1.953 | 5.467 |
| 1990–1994 | 3.208 | 1.449 | - | 4.967 |
| 1995–1999 | 2.147 | 1.290 | 1.367 | 3.785 |
| 2000–2004 | 2.746 | 1.907 | 1.518 | 4.813 |
| 2005–2009 | 2.494 | 1.100 | 2.686 | 3.695 |
| 2010–2014 | 2.224 | 1.526 | 1.546 | 3.600 |
| 2015–2019 | 2.417 | 1.757 | 1.619 | 3.874 |
| 2020–2024 | 2.520 | 1.565 | 1.994 | 4.002 |

Note: “–” indicates that data were not available.

TABLE S4 Changes in carbohydrate concentrations in the three major staple crops in China from 1980 to 2024.

| Period | Grain carbohydrate concentration (g/100 g) | | | |
| --- | --- | --- | --- | --- |
|  | Mean | Rice | Wheat | Maize |
| 1980–1984 | 69.251 | - | 69.251 | - |
| 1985–1989 | 65.762 | 71.340 | 60.184 | - |
| 1990–1994 | 72.121 | 76.435 | 70.729 | 69.200 |
| 1995–1999 | 69.568 | 71.514 | 67.622 | - |
| 2000–2004 | 71.600 | 76.799 | 65.785 | 72.216 |
| 2005–2009 | 67.717 | 71.878 | 62.025 | 69.249 |
| 2010–2014 | 68.687 | 76.276 | 69.285 | 60.500 |
| 2015–2019 | 70.895 | 75.517 | 66.397 | 70.770 |
| 2020–2024 | 68.738 | 74.427 | 58.750 | 73.036 |

Note: “–” indicates that data were not available.

TABLE S5 Changes in dietary fiber contents in the three major staple crops in China from 1980 to 2024.

| Period | Grain dietary fiber content (g/100 g) | | | |
| --- | --- | --- | --- | --- |
|  | Mean | Rice | Wheat | Maize |
| 1980–1984 | 0.405 | 0.405 | - | - |
| 1985–1989 | 1.505 | 0.250 | 2.760 | - |
| 1990–1994 | 1.800 | - | . | 1.800 |
| 1995–1999 | 3.514 | 1.696 | 2.633 | 6.213 |
| 2000–2004 | 2.580 | 3.081 | 0.975 | 3.683 |
| 2005–2009 | 2.674 | 0.684 | - | 4.663 |
| 2010–2014 | 7.650 | 3.300 | 12.000 | - |
| 2015–2019 | 5.864 | 3.821 | 8.958 | 4.813 |
| 2020–2024 | 6.325 | 2.973 | 12.706 | 3.297 |

Note: “–” indicates that data were not available.

TABLE S6 Changes in moisture contents in the three major staple crops in China from 1980 to 2024.

| Period | Grain moisture content (g/100 g) | | | |
| --- | --- | --- | --- | --- |
|  | Mean | Rice | Wheat | Maize |
| 1980–1984 | 15.000 | - | 15.000 | - |
| 1985–1989 | 14.114 | 14.114 | - | - |
| 1990–1994 | 13.093 | 12.285 | 12.893 | 14.100 |
| 1995–1999 | 12.428 | 11.155 | 13.700 | - |
| 2000–2004 | 11.819 | 12.314 | 12.408 | 10.736 |
| 2005–2009 | 11.845 | 12.261 | 12.124 | 11.151 |
| 2010–2014 | 11.389 | 11.869 | 10.908 | - |
| 2015–2019 | 11.013 | 13.010 | 10.000 | 10.028 |
| 2020–2024 | 12.786 | 13.173 | 12.743 | 12.443 |

Note: “–” indicates that data were not available.
